# Larvicidal Action of Cannabidiol Oil and Neem Oil against Three Stored Product Insect Pests: Effect on Survival Time and in Progeny

**DOI:** 10.3390/biology9100321

**Published:** 2020-10-01

**Authors:** Spiridon Mantzoukas, Aristeidis Ntoukas, Ioannis Lagogiannis, Nikolaos Kalyvas, Panagiotis Eliopoulos, Konstantinos Poulas

**Affiliations:** 1 Department of Pharmacy, University of Patras, 26504 Patras, Greece; ntoukasaris7@gmail.com (A.N.); lagoipp@gmail.com (I.L.); nikolaoskalyvas3@gmail.com (N.K.); 2 Department of Agrotechnology, University of Thessaly, 41500 Larissa, Greece; eliopoulos@uth.gr

**Keywords:** biopesticides, stored pests, progeny, survival time, IPM, lab tests

## Abstract

**Simple Summary:**

Each year, agricultural produce suffers significant loss and quality deterioration upon infestation by stored product insects. Synthetic insecticides represent a ready-to-go, effective pest control solution, albeit with several environmental and health risks. In this light and within the framework of integrated pest management research, the present study focuses on the potential insecticidal effect of two essential oils, neem oil and CBD, against 4th instar larvae of the harmful *Tribolium confusum*, *Oryzaephilus surinamensis* and *Plodia interpunctella*, on wheat, rice and corn seeds. CBD, especially, has been under-researched in this regard. Treatment efficacy was expressed as larval mortality in relation to dosage, time exposure intervals and product. In comparison with the control, the results showed clear dose-dependent pesticidal activity for both oils, expressed as significant larval mortality at high dose application, as high as 100% at the highest dose (90 mg/mL). Moreover, the overall survival time of the tested larvae was also considerably shorter than that of control larvae, while the treatments also produced considerably fewer offspring in the tested insects. Our results reinforce the prospect of incorporating botanical insecticides in Integrated Pest Management programs.

**Abstract:**

Stored product pests can be detrimental to agricultural produce. As much as chemical pesticides are effective control agents, they involve several environmental and health risks. Within the framework of studies on alternative pest management methods, interest has focused on a plethora of plants whose extracts have demonstrated promising action as insecticides. *Azadirachta indica* and the derived neem oil have been extensively tested against many harmful insect species. In contrast, *Cannabis sativa* L. and its main compound, CBD, a highly concentrated cannabinoid, have not been investigated much. The present study examined the potential insecticidal activity of CBD and neem oils against 4th instar larvae of *Tribolium confusum*, *Oryzaephilus surinamensis* and *Plodia interpunctella* on wheat, rice and corn seeds. Treatment efficacy was expressed in terms of larval mortality. Mortality was observed in relation to dosage, time exposure intervals and product types. The results showed clear pesticidal activity for both oils, which at high doses induced significant mortality. The treatments produced significantly fewer offspring in the insect species tested than the control. The efficacy of treatment in progeny suppression was, as expected, dose dependent.

## 1. Introduction

Insect pests are among the common factors which damage agricultural production every year, including stored grains. Insect pests of stored grains can be distinguished between the primary pests which damage intact grain kernels, and the secondary pests which do not damage the grain kernels but can cause heating of the grain and its diminished market value. The main secondary pests of corn and wheat are the confused flour beetle, *Tribolium confusum* Jacquelin du Val (Coleoptera: Tenebrionidae), the sawtoothed grain beetle, *Oryzaephilus surinamensis* (L) (Coleoptera: Silvanidae) and the Indian meal moth, *Plodia interpunctella* (Hϋbner) (Lepidoptera: Pyralidae) [1]. In the context of Integrated Pest Management (IPM) for stored products, prevention strategies are much more important than curative ones for both types of pests, because the arthropods that die from the application of the latter methods and remain in the stored materials are still a phytosanitary problem.

Prior to synthetic pesticides, plant extracts were the principal means against arthropod pests, because they produce powerful chemicals with repellent and/or insecticidal properties. Their effectiveness has been shown to vary depending on the dose used and the targeted arthropods [2,3,4,5,6,7,8,9].

A century ago, synthetics came to dominate the market because of their greater efficacy, longer duration of action, and more stable shelf life in comparison with plant-based products. While this may be true, the widespread use of synthetic pesticides has caused considerable damage to worldwide ecosystems, and it has also polluted the air, the water, and the soil. Synthetic pesticides may be harmful to non-target species, and directly toxic to users [3,10]. Widespread usage has also led to the development of resistance among target species [11].

Residual insecticides and fumigants are the principal contemporary means for the control of stored product pests. Despite success in controlling insect pests using synthetic insecticides [12], their persistence in the environment, the toxic residues they leave in food and the development of resistance by insect pests require that more reduced-risk alternatives are sought [13].

In general terms, plant-based biopesticides can be produced in a sustainable manner as they are inexpensive to extract, nonirritating to the skin, and considered natural [14]. They are culturally acceptable in communities with a tradition of plant use, and they are gaining popularity as substitutes for synthetic pesticides. The exploitation of hemp by-products as a source of botanical insecticides is a matter of interest for farmers, allowing them to maximize the commercial value of hemp cultivation [15].

Several plant species, such as *Azadirachta indica* A. Juss (Sapindales: Meliaceae)*, Melia azedarach* L. (Sapindales: Meliaceae), *Lantana camara* L. (Lamiales: Verbenaceae, *Eucalyptus* spp. (Myrtaceae: Myrtoideae), *Solanum nigrum* L. (Solanales: Solanaceae), and *Origanum vulgare* L. and *Thymus vulgaris* L. (Lamiales: Lamiaceae), are known to possess insecticidal properties [16,17,18], although only a few have been exploited commercially. The compounds of these plants have a series of useful anti-insect properties such as toxicity, repellence, feeding and oviposition deterrence and insect growth regulative activity [19].

Recently, there has been a growing interest for *Cannabis sativa* L. (Rosids: Cannabaceae) and its extracts for many medicinal and commercial purposes [20,21]. Spatial repellents derived from *C. sativa* were always deployed against human pests [22,23]. Targets included mosquitoes, fleas, lice, ticks, bedbugs, and scabies mites. Cannabis-based insecticides and repellents were also traditionally employed to protect crops from phytophagous arthropods [24]. The most promising substance in this plant is cannabidiol (CBD) oil, which seems to show some antimicrobial, antioxidant and other activities according to some surveys [24,25,26].

Additionally, much scientific research has been conducted on the neem tree [27,28,29,30,31,32,33,34,35,36,37,38]. The extracted oil shows pharmaceutical, anticancer, antioxidant and other properties according to many recent studies [27,28,29,30,31,32]. There are also neem oil-based products, which are developed to enhance these healing and protective properties [30,33]. Finally, this substance may show pesticidal activity against certain insect species, as has been reported in some publications [27,34,35,36,37,38].

Our idea is to obtain bioactive essential oils from the inflorescences of industrial hemp that usually remain underutilized, in order to manufacture natural insecticides for application in organic agriculture and IPM programs. Indeed, research on this issue is still poor. The objective of the present study was to evaluate the larvicidal effect of CBD oil and neem oil in vitro, against the three major stored product pests, *T. confusum, O. surinamensis* and *P. interpunctella* in three different seeds. The potential insecticidal properties of these oils might lead to an increase in pest control efficiency and improve pest management strategies.

## 2. Methods

### 2.1. Insect Rearing

Three major stored product pests were tested in the present study: *O. surinamensis*, *T. confusum* and *P. interpunctella*. *O. surinamensis* was reared on whole oats, *T. confusum* on a mixture of whole wheat flour and dried yeast (1:10), and *P. interpunctella* was provided with a mixture of honey, glycerin, dried yeast, and sterile fully-grinded flour and wheat bran. The insect colony used was established in 2018 from a stock colony provided by the University of Thessaly, Department of Agrotechnology, Laboratory of Entomology (Larisa, Greece), and it has since then been continuously grown in the Laboratory of Molecular Biology and Immunology, Department of Pharmacy, University of Patras. Insects were placed with 200–250 g of their diet in glass jars (0.45 L capacity) which were then covered with a sterilized muslin cloth. Two weeks later, we carefully sieved out the adult individuals. Insects were contained in a growth chamber (PHC Europe Sanyo Panasonic Biomedical MLR-352-PE), in controlled environmental conditions, at 27.5 °C and 75% R.H.

### 2.2. Bioassay

The experiments were carried out at the Laboratory of Molecular Biology and Immunology, Department of Pharmacy, University of Patras. The CBD oil obtained from the company Elixinol, Enecta CBD oil (Athens, Greece), contains 3% CBD. The neem oil acquired from Laboratoire Alth, Organic neem Oil (Monfort, France) contains 3% azadirachtin. MeOH (1%) was used for the dilution of both oils; the dilution process was carried out inside a laminar flow chamber (Equip Vertical Air Laminar Flow Cabinet Clean Bench, Mechanical Application LTD, Athens, Greece). We tested the effectiveness of the oils against larvae of *T. confusum, O. surinamensis* and *P. interpunctella*, at three different doses, 90 mg/mL (hi con), 45 mg/mL (med con), 15 mg/mL (low con). The experimental product was directly sprayed with 2 mL using a Potter spray tower (Burkard Manufacturing Co. Ltd., Rickmansworth, Hertfordshire, U.K.) at 1 kgf cm^−2^. After applying the CBD and Neem oils, the product lots were placed back in the petri dish, and they were shaken manually for 30 s to achieve equal distribution of the oil; the product was then air dried for 30 min. The tests were carried out on wheat cv. Mexa which contained 5% cracked kernels, corn cv. Golden Bantam which contained 5% cracked kernels, and rice cv. Japonica which also contained 5% cracked kernels. These products were adjusted at 12% moisture content (m.c.), via storage in ambient conditions for 28 d.

Laboratory-reared 4th instar larvae from each experimental insect species were used for this study. Each batch of ten 4th instar larvae was collected from the rearing jars and then placed in 9-cm diameter petri dishes with 10 g of the desirable product, after the larvae had been starved for 1 h. Ten larvae were used per replication (n = 10), and ten replications were carried out per dose (n = 100); the experiment was replicated ten times (n = 1000). The petri dishes had a plastic lid with a hole in the center covered with fine mesh, while the internal “upper lid” of the petri dishes was covered with Fluon (Northern Products, Woonsocket, RI, USA), to prevent insect individuals from escaping. Then, all petri dishes were placed in incubators set at 27.5 °C and 75% R.H. The control larvae were treated with MeOH and ddH_2_O. Mortality of the exposed larvae was examined after 12, 24, 48, 72, 144, 216 and 288 h.

After 288 h, the petri dishes containing *T. confusum*, *O. surinamensis* and *P. interpunctella* larvae, were shielded with parafilm and placed back in the incubators, in the same conditions, for an additional period of 25 d. After the termination of this interval, the petri dishes were opened, and progeny was recorded. Progeny was recorded separately per life stage of larvae or pupae.

### 2.3. Statistical Analysis

Preceding analysis, we arcsin transformed all values. We analyzed data by five-way ANOVA with the general linear model of the SPSS (SPSS Inc., Armonk, NY, USA, version 25) (IBM, 2019), and we compared means of significant F values with the Bonferroni test. We also applied the Kaplan–Meier method to determine the mean survival time of *T. confusum, O. surinamensis* and *P. interpunctella* larvae post treatment application.

The percentage of pupal and adult inhibitions for *P. interpunctella, T. confusum* and *O. surinamensis* is expressed as equal to:

PI or AI = 100 ∗ (1 − t / c)

whereby t denotes the number of pupae or adults in the treatment, and c refers to pupae or adults in the control. We processed data with one-way ANOVA, using the general SPSS linear model (SPSS Inc., Armonk, NY, USA, version 25) (IBM, 2019), and means of significant F values were compared with the Bonferroni test.

## 3. Results

### 3.1. Larvicidal Effect of CBD Oil and Neem Oil on Stored Pest in Vitro Assay

Significant differences were detected among treatments in many cases. Oil Dose, Insect Species, Seeds Product and Time were proven to have a significant effect on larval mortality. Factors interactions showed a significant effect; this is indicative of the fact that experimental factors affected the insects’ survival time in diverse ways (Table 1). Very low control mortality was recorded among all experimental larvae on all products. Mean control mortality was recorded between 3.33% and 6.67% in all cases.

**Figure 1:**
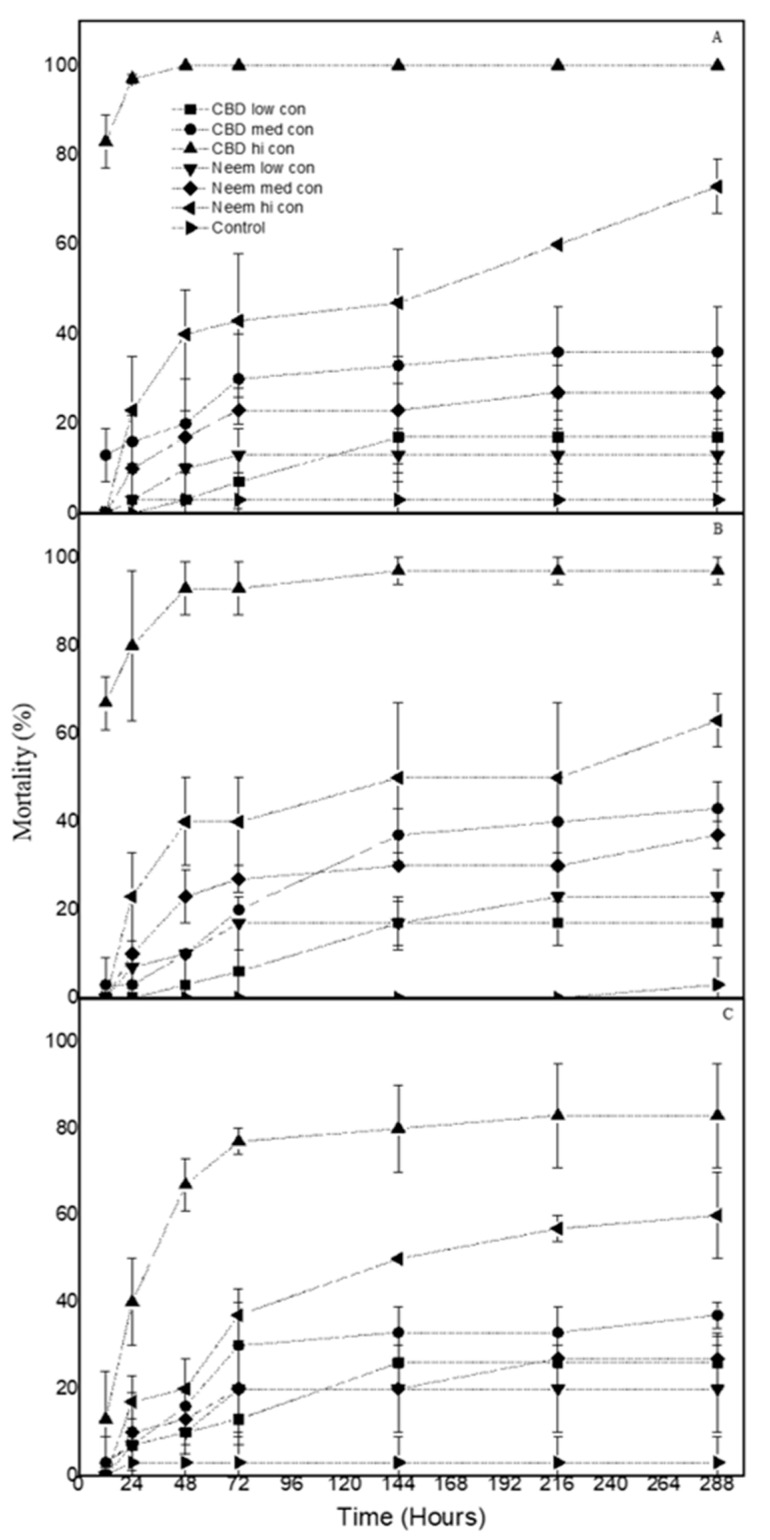
Mean mortality (% ± sd) of *T. confusum* larvae treated with CBD oil and neem oil at three concentrations on (**A**) wheat (**B**) corn and (**C**) rice. (low con: 15 mg/mL, med con: 45 mg/mL, hi con: 90 mg/mL).

When *T. confusum* larvae were exposed to the CBD oil, they suffered mortality between 17% and 100% on wheat, 17% and 93% on corn, and 26% and 83% on rice. Similarly, the neem oil caused larval mortality between 13% and 73% on wheat, 23% and 63% on corn, and between 20% and 60% on rice (Figure 1). In all cases, mortality was always significantly increased as the dose increased.

**Figure 2:**
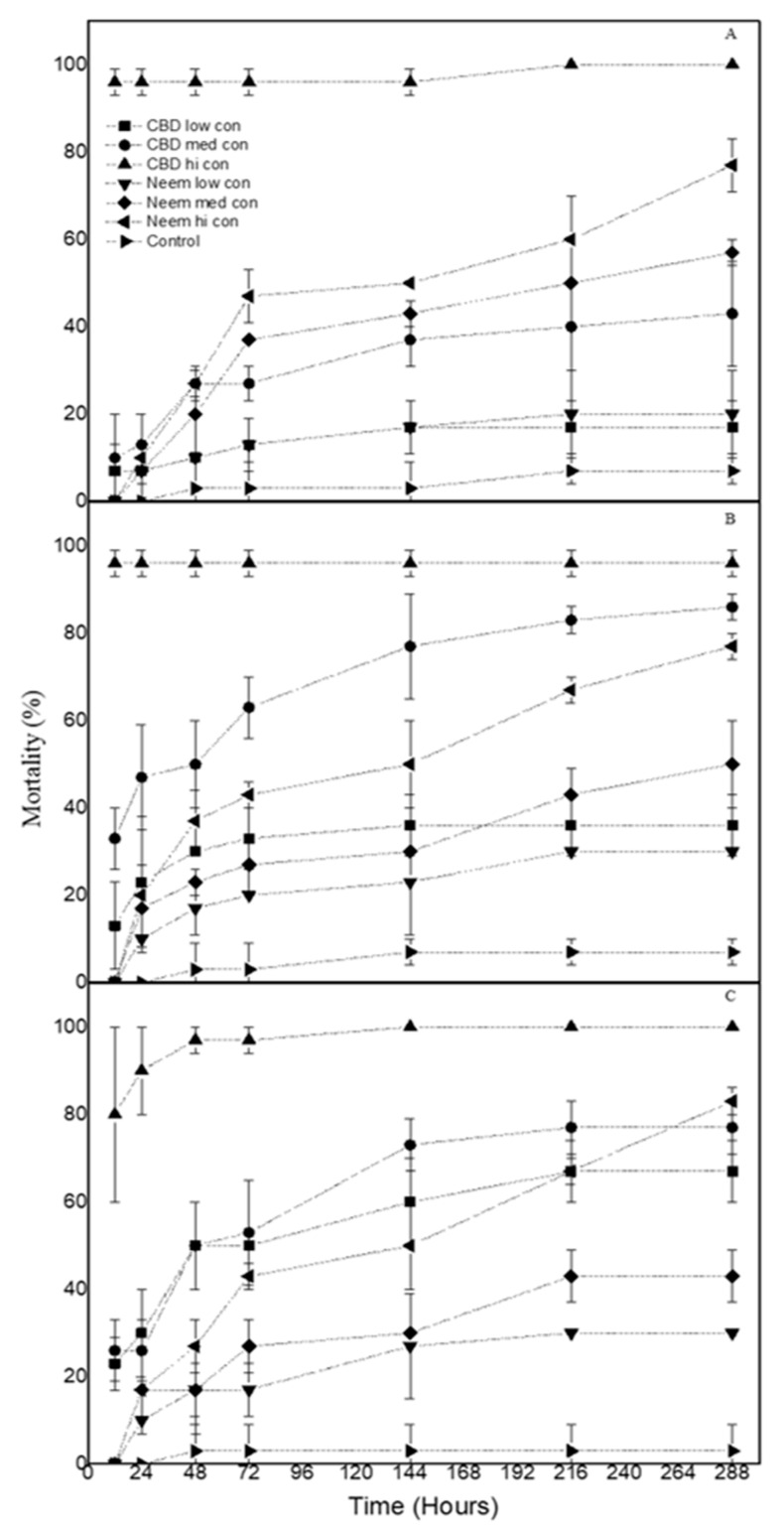
Mean Mortality (% ± sd) of *O. surinamensis* larvae treated with CBD oil and neem oil at three concentrations on (**A**) wheat (**B**) corn and (**C**) rice. (low con: 15 mg/mL, med con: 45 mg/mL, hi con: 90 mg/mL).

As far as *O. surinamensis* larvae are concerned, their mortality ranged between 17% and 100% on wheat, 36% and 96% on corn, and 67% and 100% on rice, when they were sprayed with CBD oil. The respective neem oil treatments caused larval mortality between 20% and 77% on wheat, 30% and 77% on corn, and 30% and 83% on rice (Figure 2).

**Figure 3:**
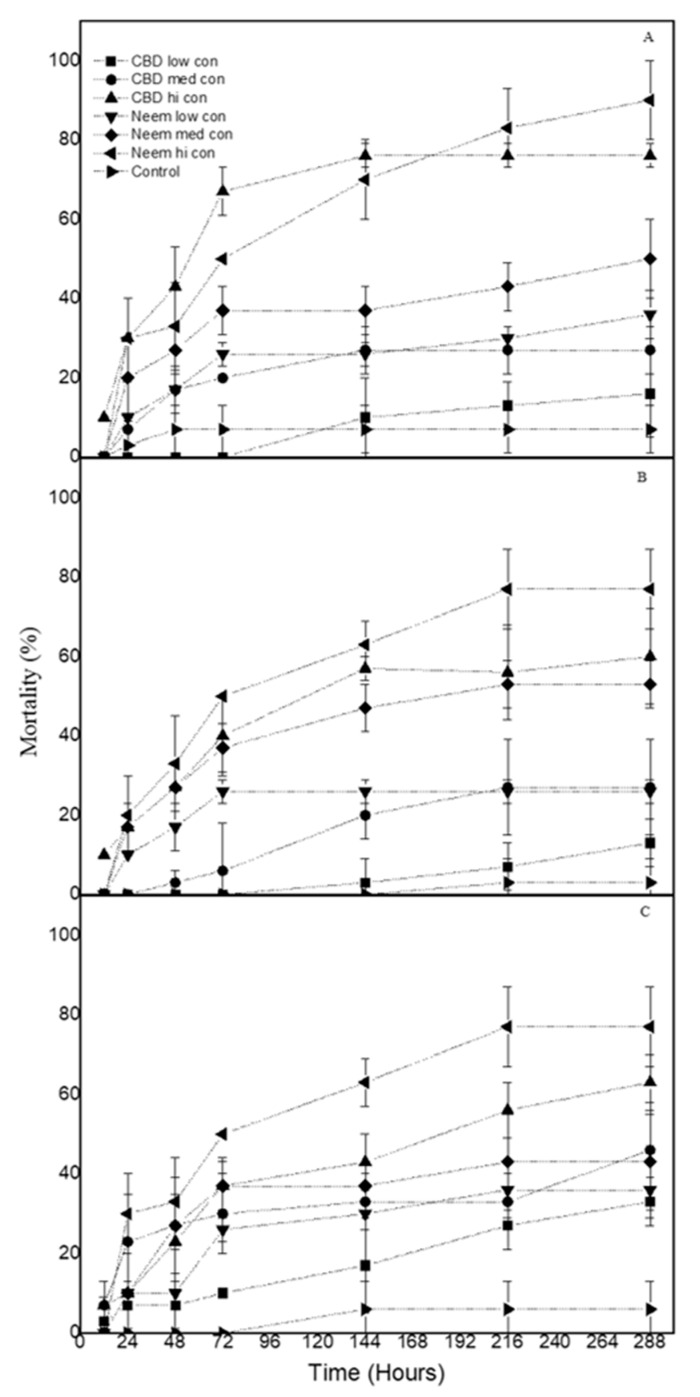
Mortality (% ± sd) of *P. interpunctella* larvae treated with CBD oil and neem oil at three concentrations on (**A**) wheat (**B**) corn and (**C**) rice. (low con: 15 mg/mL, med con: 45 mg/mL, hi con: 90 mg/mL).

Finally, the mortality of *P. interpunctella* larvae treated with CBD oil was between 16% and 76% on wheat, 13% and 60% on corn, and 33% and 63% on rice. Accordingly, the neem oil caused larval mortality between 36% and 90% on wheat, 26% and 77% on corn, and 36% and 77% on rice (Figure 3).

### 3.2. Overall Survival Time of Larvae Exposed to Doses of CBD Oil and Neem Oil

The survival time of *T. confusum* larvae exposed at overall low, mid and high doses was recorded at 222.667, 159.971 and 63.067 h. Similarly, the survival time of *O. surinamensis* larvae exposed at overall low, mid and high doses was recorded at 245.667, 146.933 and 58.000 h, respectively (Table 2). Finally, the survival time of *P. interpunctella* larvae exposed at overall low, mid and high doses was recorded at 244.267, 196.0000 and 87.333 h, respectively. Significant differences were detected between insect and dose (Table 2).

The overall survival time of larvae exposed to the CBD oil at the low, mid and high doses was recorded at 219.156, 180.556 and 75.956 h. Similarly, the overall survival time of larvae exposed to the neem oil at the low, mid and high doses was recorded at 239.244, 204.044 and 119.644 h. Significant differences were detected between both treatments with the control and between the high dose of CBD and the high dose of the neem oil (Table 3).

### 3.3. Effect of CBD Oil and Neem Oil Doses to Pupation and Adult Emergence

Results related to inhibition of metamorphosis showed that a noticeable decrease in percentage achievement of pupal and adult stage was detected when 4th stage larvae of *P. interpunctella*, *T. confusum* and *O. surinamensis* were subjected to high doses of CBD oil and neem oil. At the higher dose of CBD oil, the lowest pupation and adult emergence were 8.43% and 6.13% for *P. interpunctella* (Table 4); 5.75% and 3.13% for *T. confusum* (Table 5); and 3.71% and 1.89% for *O. surinamensis* (Table 6). In parallel, at the higher dose of neem oil the lowest pupation and adult emergence were 10.10% and 9.08% for P. interpunctella (Table 4); 7.10% and 6.04% for *T. confusum* (Table 5); and 4.10% and 1.13% for *O. surinamensis* (Table 6).

By contrast, at the lower doses of the CBD oil, the highest pupation and adult emergence were 30.12% and 26.90% for *P. interpunctella* (Table 4); 25.12% and 21.90% for *T. confusum* (Table 5); and 26.19% and 24.08% for *O. surinamensis* (Table 6). Similarly, at the lower dose of neem oil, the highest pupation and adult emergence were 29.87% and 28.20% for *P. interpunctella* (Table 4); 26.87% and 24.19% for *T. confusum* (Table 5); and 28.14% and 25.90% for *O. surinamensis* (Table 6).

## 4. Discussion 

Essential oils have been successfully used to manage a rather wide number of serious insect pests [39,40]. The present study has highlighted substantial insecticidal activity of CBD oil and neem oil against *T. confusum*, *O. surinamensis* and *P. interpunctella* larvae and pupae, as high as 100% at the highest dose (90 mg/mL), whereby the overall survival time of the tested larvae was also considerably shorter than that of control larvae. 

Pavela [41] reported on the significant aphicidal efficacy of CBD oil against *Myzus persicae* Sϋlzer (Hemiptera: Aphididae), as well as on its significant toxicity against *Musca domestica* L. (Diptera: Muscidae)*, M. persicae, Culex quinquefasciatus* Say (Diptera: Culicidae) and *Spodoptera littoralis* Boisduval (Lepidoptera: Noctuidae) larvae [42]. Sharma et al. [43] and Sharma et al. [44] assayed toxicidal activity of CBD oil against *Plutella xylostella* L. (Lepidoptera: Plutellidae) and *Phthorimaea operculella* Zeller (Lepidoptera: Gelechiidae). The present study, which represents the first report on the lethal effect of CBD oil on *T. confusum*, *O. surinamensis* and *P. interpunctella*, reiterates the value of CBD oil as a larvicide.

Similarly, neem oil caused high mortality to all three insects as well as low survival time when administered at the highest dose. This adds to the existing bibliography on the role of neem oil in providing sustained protection of stored grains [45] and efficient pest control in storage facilities, alone or in combination with other protective measures [46,47,48,49,50,51,52,53,54,55]. Only a few studies have investigated the effect of neem oil on the survival of our experimental insect species. Indicatively, it has been found that neem extracts have caused noteworthy mortality to *T. confusum* [56,57] and *O. surinamensis* adults [58], whereas no data have been published on *P. interpunctella*. 

Moreover, the two oils were able to suppress progeny production and adult emergence in all tested species. A similar effect has been reported by Paranagana et al. [59] about the essential oils of lemongrass (*Cymbopogon citratus* Stapf (Poales: Poaceae)), citronella (*Cymbopogon nardus* Stapf (Poales: Poaceae)), and cinnamon (*Cinnamomum zeylanicum* J.Presl (Laurales: Lauraceae)) which deterred oviposition and suppressed progeny production of the cowpea weevil *Callosobruchus maculatus* Fabricius (Coleoptera Chrysomelidae).

## 5. Conclusions

In general terms, both oils displayed noteworthy action against the tested larvae of *P. interpunctella, T. confusum* and *O. surinamensis*. As such, our results allow us to state that these stored product pests and the larvae of *O. surinamensis,* exhibit a clear susceptibility to the tested oils, which warrants further research. As the target of limiting the use of synthetic pesticides becomes more urgent, the prospect of incorporating botanical insecticides in Integrated Pest Management programs comes to the forefront.

## Figures and Tables

**Table 1:** ANOVA parameters for main effects and associated interactions between Product, Hours, Dose, Insect larvae and Treatment, over the Dependent Variable of Mortality. Non-significant interactions have been omitted for brevity.

Factor	df	F	Sig.
Product	2	1.032	0.000
Time	6	24.988	0.000
Oil Dose	3	116.531	0.000
Insect Species	2	6.632	0.001
Plant Oil	1	7.019	0.000
Product * Insect Species	4	2.695	0.030
Time * Oil Dose	18	16.254	0.000
Time * Insect Species	12	11.030	0.000
Time * Plant Oil	6	23.330	0.000
Oil Dose * Insect Species	6	2.354	0.029
Product * Time * Insect Species	24	2.762	0.000
Time * Oil Dose * Insect Species	36	5.023	0.000
Time * Oil Dose * Plant Oil	18	10.941	0.000
Time * Insect Species * Plant Oil	12	5.626	0.000
Product * Time * Oil Dose * Insect Species	72	1.803	0.000
Product * Time * Oil Dose * Plant Oil	36	1.566	0.019
Time * Oil Dose * Insect Species * Plant Oil	36	2.740	0.000
Error	4007	0.007	
Total	4511		
Corrected Total	4510		

* Indicate the interaction.

**Table 2:** Overall survival time of larvae in relation to dose and insect (Chi-square: 51.187, df = 1, *p* < 0.001). Means of the same column followed by the same letter are not significantly different (Long Rank test, *p* = 0.05).

Dose	Insect	Estimate (Hours)	Std Deviation	95% Confidence Interval	
				Lower Bound	Upper Bound
Low	*T. confusum*	222.667 ^c^	7.812	207.354	237.979
	*O. surinamensis*	225.667 ^c^	4.496	216.854	234.479
	*P. interpunctella*	244.267 ^b^	5.819	232.862	255.671
Mid	*T. confusum*	159.971 ^d^	9.075	142.184	177.757
	*O. surinamensis*	146.933 ^d^	7.678	145.885	175.982
	*P. interpunctella*	196.000 ^e^	7.602	185.101	216.899
High	*T. confusum*	63.067 ^g^	5.507	42.273	83.860
	*O. surinamensis*	58.000 ^g^	8.255	41.820	64.180
	*P. interpunctella*	87.333 ^f^	6.485	64.623	100.044
Control	*T. confusum*	272.133 ^a^	6.412	259.565	284.702
	*O. surinamensis*	281.600 ^a^	2.610	276.484	286.716
	*P. interpunctella*	286.400 ^a^	0.646	285.134	287.666

**Table 3:** Overall survival time of larvae in relation to dose and treatment (Chi-square: 18.646, df = 3, *p* < 0.001). Means of the same column followed by the same letter are not significantly different (Long Rank test, *p* = 0.05).

Dose	Treatment	Estimate (Hours)	Std Deviation	95% Confidence Interval	
				Lower Bound	Upper Bound
Low	*CBD oil*	219.156 ^b^	5.600	218.179	240.132
	*Neem oil*	239.244 ^c^	4.701	250.030	268.459
Mid	*CBD oil*	180.556 ^e^	7.069	166.700	184.412
	*Neem oil*	204.044 ^d^	6.694	190.925	217.164
High	*CBD oil*	75.956 ^g^	6.187	63.829	88.082
	*Neem oil*	119.644 ^f^	6.162	107.568	131.721
Control		284.800 ^a^	1.346	282.162	287.438

**Table 4:** Mean effect of CBD oil and neem oil on pupation and adult emergence (% ± sd) of 4th larval instars of *P. interpunctella.* Means of the same column followed by the same letter are not significantly different (Long Rank test, *p* = 0.05).

Dose	Treatment	Pupation (% ± sd) (F = 81.52 df = 16, *p* < 0.001)	Adult Emergence (% ± sd) (F = 15.88, df = 16, *p* < 0.001)
Low	*CBD oil*	30.12 ± 1.39 ^b^	26.90 ± 1.93 ^b^
	*Neem oil*	29.87 ± 2.12 ^b^	28.20 ± 1.03 ^b^
Mid	*CBD oil*	25.04 ± 2.52 ^c^	22.13 ± 1.01 ^d^
	*Neem oil*	29.24 ± 0.89 ^d^	26.08 ± 2.63 ^c^
High	*CBD oil*	8.43 ± 0.60 ^f^	6.13 ± 4.13 ^e^
	*Neem oil*	10.10 ± 0.62 ^e^	9.08 ± 3.50 ^e^
Control		92.81 ± 1.98 ^a^	86.10 ± 2.10 ^a^

**Table 5:** Mean effect of CBD oil and neem oil on pupation and adult emergence (% ± sd) of 4th larval instars of *T. confusum*. Means of the same column followed by the same letter are not significantly different (Long Rank test, *p* = 0.05).

Dose	Treatment	Pupation (% ± sd) (F = 81.52 df = 16, *p* < 0.001)	Adult Emergence (% ± sd) (F = 10.34, df = 16, *p* < 0.001)
Low	*CBD oil*	25.12 ± 1.49 ^c^	21.90 ± 0.93 ^c^
	*Neem oil*	26.87 ± 2.52 ^c^	24.19 ± 1.23 ^c^
Mid	*CBD oil*	17.04 ± 0.89 ^b^	15.13 ± 1.11 ^b^
	*Neem oil*	19.04 ± 1.89 ^c^	21.08 ± 1.63 ^c^
High	*CBD oil*	5.75 ± 2.20 ^e^	3.13 ± 2.13 ^e^
	*Neem oil*	7.10 ± 2.62 ^d^	6.04 ± 2.50 ^d^
Control		89.81 ± 1.98 ^a^	86.10 ± 2.10 ^a^

**Table 6:** Mean effect of CBD oil and neem oil on pupation and adult emergence (% ± sd) of 4th larval instars of *O. surinamensis.* Means of the same column followed by the same letter are not significantly different (Long Rank test, *p* = 0.05).

Dose	Treatment	Pupation (% ± sd) (F = 70.24 df = 16, *p* < 0.001)	Adult Emergence (% ± sd) (F = 9.11, df = 16, *p* < 0.001)
Low	*CBD oil*	26.19 ± 1.85 ^b^	24.08 ± 0.93 ^b^
	*Neem oil*	28.14 ± 1.92 ^c^	25.90 ± 2.23 ^b^
Mid	*CBD oil*	19.51 ± 2.54 ^b^	12.13 ± 1.11 ^c^
	*Neem oil*	20.19 ± 1.85 ^c^	17.19 ± 1.79 ^c^
High	*CBD oil*	3.71 ± 2.23 ^d^	1.89 ± 2.63 ^d^
	*Neem oil*	4.10 ± 1.92 ^d^	1.13 ± 2.13 ^d^
Control		91.07 ± 1.98 ^a^	88.54 ± 2.10 ^a^
